# Dielectric Characterization of Fabric Aggregates around the 2.45 GHz ISM Band under Various Humidity, Density, and Temperature Conditions

**DOI:** 10.3390/ma16124428

**Published:** 2023-06-16

**Authors:** Rafael Pérez-Campos, Juan Monzó-Cabrera, José Fayos-Fernández, Alejandro Díaz-Morcillo, Antonio Martínez-González, Antonio José Lozano-Guerrero, Juan Luis Pedreño-Molina, Jose Antonio García-Gambín

**Affiliations:** Departamento de Tecnologías de la Información y las Comunicaciones, Universidad Politécnica de Cartagena, 30202 Cartagena, Spain; rafael.perez@upct.es (R.P.-C.); jose.fayos@upct.es (J.F.-F.); alejandro.diaz@upct.es (A.D.-M.); toni.martinez@upct.es (A.M.-G.); antonio.lozano@upct.es (A.J.L.-G.); juan.pmolina@upct.es (J.L.P.-M.); jose.jagg11@gmail.com (J.A.G.-G.)

**Keywords:** permittivity, fabric aggregates, cotton, polyester, polyamide

## Abstract

Fabric permittivity is critical for the manufacturing of wearable sensors and antennas as well as predicting how fabrics interact with electromagnetic fields. Engineers should also understand how permittivity changes under different temperatures, densities, and moisture content values, or when several fabrics are mixed in aggregates, when designing future applications such as microwave dryers. The permittivity of cotton, polyester, and polyamide fabric aggregates is investigated in this paper for a wide range of compositions, moisture content levels, density values, and temperature conditions around the 2.45 GHz ISM band using a bi-reentrant resonant cavity. The obtained results show extremely comparable responses for all characteristics investigated for single and binary fabric aggregates. Permittivity always increases as temperature, density, or moisture content levels rise. Moisture content is the most influential characteristic, causing enormous variations in the permittivity of aggregates. Fitting equations are supplied for all data, with exponential functions used to accurately model variation in temperature and polynomial functions employed to precisely model density and moisture content variations with low error levels. The temperature permittivity dependence of single fabrics without the influence of air gaps is also extracted from fabric and air aggregates by using complex refractive index equations for two-phase mixtures.

## 1. Introduction

Fabrics are washed and dried on a regular basis, which takes a lot of time and energy. Traditional tumble dryers use hot air to remove moisture from fabric aggregates, which involve various types of fabrics that are loosely mixed, but the time required is long due to the low thermal conductivity of fabrics. Because of the interaction of the electric field with water dipolar molecules, which rapidly generates heat within the fabrics, microwave drying can significantly reduce fabric drying times without sacrificing material quality [1]. Furthermore, ref. [2] demonstrates that combining microwave drying and heat recovery can result in significantly higher efficiency levels than traditional clothes dryers. Despite the large number of patents in this field, commercial microwave dryers have yet to reach consumers due to substantial technical difficulties in designing and manufacturing these devices [3].

Fabric aggregates can be made from a variety of materials, and when dried in rotating drums, they can exhibit different densities as well as high temperature and moisture content variations over time, which makes predicting their permittivity during the drying process one of the technical challenges for the design of microwave dryers.

Permittivity provides useful information about the rate at which the material heats up when microwaves are applied, as well as the distribution of the electric field within the material. The relative permittivity, $\varepsilon_{r}^{*}$, which is normalized versus vacuum permittivity, is a complex characteristic with a real part, often called the dielectric constant, $\varepsilon_{r}^{\prime }$, and an imaginary part, $\varepsilon_{r}^{\prime \prime }$, known as the loss factor: $\varepsilon_{r}^{*}=\varepsilon_{r}^{\prime }-j\varepsilon_{r}^{\prime \prime }$. Both the dielectric constant and the loss factor of fabrics are affected by frequency, moisture content, density, and temperature.

Several techniques can be used to perform the dielectric characterization of fabric aggregates, including parallel plate, resonant cavity, coaxial probe, transmission line, and free space techniques [4]. These methods enable the measurement of dielectric properties over a wide range of frequency ranges and under varying moisture, density, and temperature conditions.

Yamada presents an in-depth examination of the dielectric properties of textile materials, including measurement, analysis methodologies, and the prospective applications of fabrics in microwave engineering [5]. Another new area that has gained increasing interest in the literature is the investigation of the dielectric characteristics of various fabrics for wearable antennas versus fabric thickness and composition [6,7,8]. Other authors in [9,10,11] investigate the effects of humidity and temperature on fabric permittivity. Methods to measure textile materials’ complex permittivity at microwave frequencies have also been presented, and their potential applications in the design of textile-based microwave devices have been described in [8,12].

Dielectric aggregates, on the other hand, are substances made from a mass of pieces or particles with various dielectric constants that are loosely packed together. This is the typical configuration in which clothing is combined in tumble dryers and, therefore, determining the permittivity of fabric aggregates is essential to predict the behavior of multimode cavities during microwave drying. Tuncer et al. [13] provided an overview of dielectric mixture properties and their modeling. Other studies of dielectric aggregates can be found in several research areas such as the production of biofuels [14] or in mixes of mineral and vegetable oils [15]. In ref. [16], Nelson examined two techniques, which were previously outlined in [17,18,19,20,21], for permittivity data extrapolation and computation using dielectric mixing equations. It is also explained in those works how to calculate the permittivity of solids using data on measured permittivity obtained from granular or powdered samples.

This article addresses the dielectric characteristics of fabric aggregates for a variety of composition, moisture content, density, and temperature conditions using a bi-reentrant resonant cavity around the 2.45 GHz Industrial, Scientific, and Medical (ISM) band so that future researchers can design and implement microwave drying devices for these materials, since those devices will deal directly with those aggregates. Because the designers of wearable antennas and other types of microwave devices that will be embedded in those fibers may find this information useful, the influence of air in the tests is also eliminated by using the complex refractive index (CRI) mixing equations to estimate the permittivity of several fabrics under different temperature conditions from the measurements. For measurements of single-material fabric aggregates, as well as binary combinations of cotton, polyester, and polyamide aggregates, large changes in permittivity versus temperature, density, and, especially, moisture content have been observed.

## 2. Materials and Methods

### 2.1. Dielectric Measurement Technique

**Figure 1:**
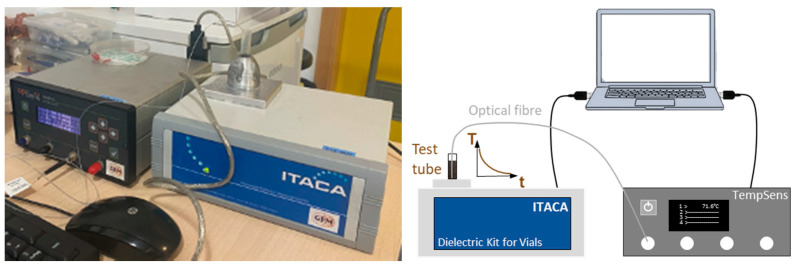
Permittivity measurement set-up with the DKV and simultaneous temperature measurement of the sample (**left**) and associated scheme (**right**).

The permittivity measurements of fabric aggregates will result in a wide range of dielectric constant and loss factor values, especially due to moisture content variations. Thus, a technique that can handle this large permittivity range with high accuracy must be carefully chosen. This is the purpose of the combined bi-reentrant resonant cavity and de-embedding of the feeding network method, which was introduced in [22] and commercially implemented in the Dielectric Kit for Vials (DKV) from the Institute of Information and Communication Technologies (ITACA) settled in Valencia, Spain [23]. This instrument, shown in Figure 1, can measure the loss factor and dielectric constant of a variety of liquid, granular, or powdered substances at frequencies near 2.45 GHz. Specifically, it operates in the 1.5–2.6 GHz frequency region, and it can offer loss-factor values between 0.001 and 15, with an accuracy of 5%, and dielectric constant values less than 100, with an accuracy averaging approximately 1%. The manufacturer provides repeatability and linearity levels of about 0.2%. Due to the high variation of moisture content analyzed, the resonant frequencies for the measurements ranged from 2.015 to 2.542 GHz and can be extrapolated to the 2.45 GHz ISM band. When evaluating the evolution of fabric permittivity versus temperature, as illustrated in Figure 1, the DKV instrument was utilized concurrently with a TempSens fiber-optic thermometer from OpSens Solutions Inc. based in Québec, QC, Canada, even though it was employed alone to measure permittivity versus moisture content or density. The following sections provide an explanation of the various techniques for generating the fabric samples and evaluating permittivity in relation to moisture content, density, and temperature.

### 2.2. Generation of Fabric Aggregates

In order to investigate and simulate how electrical permittivity changes with the composition of clothing in the aggregate, many samples with different percentages of cotton, polyamide, and polyester were created. Three distinct types of clothing were utilized in this project: a sports shirt (94% polyamide, 6% elastane), underwear (88% polyester, 12% elastane), and an underwear T-shirt (100% cotton). Every article of clothing was bought from a well-known supermarket brand in Spain in order to use commercial fabrics that were frequently bought, washed, and dried in Spanish homes. Since the 12% and 6% elastane in the first two items were concentrated in the upper part of the underwear and the reinforcement of the T-shirt, respectively, rather than being mixed throughout, they could be easily removed. These elastane components were removed by meticulously cutting them out with scissors. We were able to measure various aggregates of polyester, polyamide, and pure cotton with this method.

The permittivity values of these textiles were evaluated for single materials (100% of the mass was for a single material) and in binary aggregates (67–33% in mass) for various densities, moisture levels, and temperatures. The basic specimens are shown in Table 1. Three permittivity samples per specimen were taken at each point of density, temperature, and moisture content. The data from the three permittivity samples were then combined to obtain the average permittivity value and its standard deviation.

**Figure 2:**
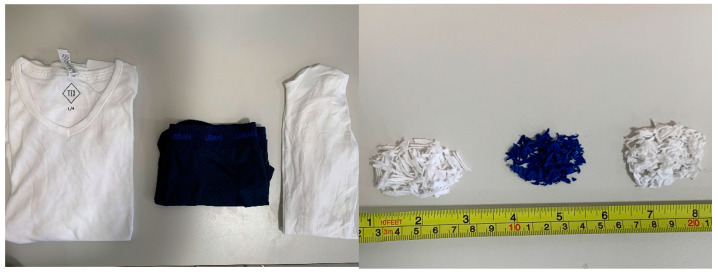
Commercial garments (**left**) and chopped fabrics (**right**) used for the obtention of the different samples.

The fabrics that made up each sample were first prepared by being chopped into pieces a few square millimeters in size. Figure 2 shows the garments (left) from which the different samples were obtained and the chopped fabrics (right) obtained from these garments. This was necessary to guarantee an equal mix and avoid significant air gaps in the sample. A tube was then filled with fabrics, and the mass of each sample was monitored until the desired mass was attained. In this study, the mass of the sample was fixed at 1.8 g for temperature and at 0.9 g for density. The mass was varied in the study of permittivity change versus moisture content, with a maximum starting value of 4.8 g. When measuring sample permittivity against temperature, the mass of the sample permitted the fiber optic thermometer to be inserted outside the DKV instrument’s cavity, preventing any disturbance in measurements. For permittivity measurements as a function of density, the chosen sample mass allowed for a proper range of volumes to be tested, yielding a wide range of bulk densities. Finally, the internal moisture content of fabrics determined the initial mass of samples in permittivity tests versus moisture content.

When making a binary mixture, both textiles were homogeneously combined before being added to the vial. The mass and volume were precisely measured for each sample, yielding identical values in the three samples of each specimen to allow the determination of the average value and the standard deviation of permittivity measurements under the same conditions.

### 2.3. Measurement Methods

#### 2.3.1. Temperature Dependence Measurements

The following method was applied to all samples in order to perform permittivity measurements versus cloth temperature: a glass of tap water was heated in a microwave oven until it reached a temperature of 90 °C. The tube containing the fabric sample and an optical thermometer were then placed in the heated water and kept there until the sample reached its maximum temperature, and the tube was quickly dried with a paper towel before being placed in the DKV.

The dielectric constant and loss factor data were obtained with the DKV [23] while the temperature was simultaneously monitored using a TempSens optical-fiber thermometer from Opsens [24]. The optical fiber sensor was not placed in the DKV cavity, thereby avoiding any disturbance in the permittivity measurements. For temperatures above 45 °C, the TempSens temperature precision is equal to 0.8 °C or better, and for temperatures below 45 °C, it is equal to 0.3 °C or better.

It was possible to relate the dielectric properties to the measured temperature data in a way similar to that described in [25,26] by comparing the temperature and permittivity time vectors. It should be mentioned that each complex permittivity value was calculated by averaging the results from three tests. The sample temperature was measured in accordance with Newton’s law of cooling, which predicts that temperature decreases more rapidly at higher temperatures; as a result, more permittivity data was collected at lower temperatures than at higher ones. To acquire permittivity values with comparable temperatures, the averaged permittivity values within 1 °C temperature bins were again averaged [25,26].

#### 2.3.2. Moisture Content Dependence Measurements

The variation of samples’ permittivity versus moisture content followed a different methodology. The chopped fabric pieces were placed in water until a 400% mass increment was detected. The DKV tubes were then filled with single fabrics or binary mixtures until a mass of 4.8 g was achieved. These filled tubes were placed in an electric oven at a constant temperature of 80 °C for 2 h. After this drying period, the tubes were extracted and left at ambient temperature until they completely cooled down. During the cooling period, the test tubes were closed to avoid additional moisture evaporation. When the samples were at ambient temperature, 25 °C, they were weighed and then placed in the DKV to obtain the permittivity values. Each permittivity value versus moisture content was the result of averaging three individual measurements. The two-hour drying and cooling periods, as well as the weight measurement and permittivity estimation, were repeated until the mass was entirely dried.

The sample moisture content, *X*, was measured on a dry basis according to Equation (1):

(1) $$X=\frac{m_{w}-m_{d}}{m_{d}}$$

where *m_w_* is the mass of the sample with some moisture content and *m_d_* is the mass of the sample after it has been fully dried. This last criterion was assumed to be reached when the mass sample remained constant following three successive weight measurements. A weighing scale from GRAM, model SV, was used to measure the mass sample with an accuracy of 0.1 g.

#### 2.3.3. Density Dependence Measurements

In this study, the variation of fabric permittivity versus density was also examined. While the volume of each sample was changed inside a volumeter with a parallax error control (4.7 ± 0.1 cm^3^ readable), the mass of each sample was maintained constant. A scale from GRAM, model SV, with a 0.1 g accuracy, was used to measure the mass sample, which was fixed at 0.9 g. The sample volume was calculated using various filling heights inside the cylindrical DKV tubes, which have a 10.3 mm inner diameter. The initial volume of all samples was around 5.4 cm^3^.

### 2.4. Data Fit for Regression Models

The curve fitting tool from MATLAB^®^ 2012b [27] was used to fit different functions to experimental permittivity data versus temperature, bulk density, and moisture content. As many materials exhibit Arrhenius behavior versus temperature changes, exponential functions were utilized to describe the permittivity progression of single fabrics and fabrics aggregates as a function of temperature [28]. Polynomial functions were used for density and moisture content to represent permittivity evolution versus those two characteristics in a manner similar to that reported in [26,29]. The suggested functions were fitted to measured data using a nonlinear least-squares fitting approach. The statistical goodness of fit was evaluated using *R-square*, which is the square of the correlation between the observed values and the predicted response values, and the Root Mean Squared Error (*RMSE*).

### 2.5. Methods for the Estimation of Temperature Dependence of Fabric Permittivity from Samples

The permittivity of fabrics made of pure cotton, polyester, and polyamide is estimated in this work by using the complex refractive index (CRI) dielectric mixture equations [16,29] for a two-phase mixture. This allowed us to calculate the permittivity of the solid material from the permittivity of fabric-air aggregates. This estimation requires knowledge of the permittivity of the air-fabric combination, its bulk density ($\rho_{m}$), and the fabric density ($\rho_{f}$). Densities for cotton, polyester, and polyamide can be found in [30,31,32] as 1.14, 1.23, and 1.14 g/cm^3^, respectively. The CRI equations allow for the extraction of the fabric’s relative permittivity from the fabric aggregate permittivity, eliminating the influence of air gaps by using Equation (2):

(2) $$\sqrt{\varepsilon_{rm}}=v_{a}\sqrt{\varepsilon_{ra}}+v_{f}\sqrt{\varepsilon_{rf}}$$

where $\varepsilon_{rm}$ is the relative permittivity of the air-fabric aggregate, $\varepsilon_{ra}$ is the air relative permittivity ($\varepsilon_{ra}=1$), $\varepsilon_{rf}$ is the permittivity of the cotton, polyester or polyamide fabric, and $v_{a}$ and $v_{f}$ are the volume fractions of air and fabrics ($v_{a}+v_{f}=1$), respectively. Both $v_{a}$ and $v_{f}$ can be obtained from the bulk density of the mixture and the fabric density ($v_{a}=\frac{\rho_{m}}{\rho_{f}}$) [29].

Equation (2) can be separated into real and imaginary parts as indicated in [29], obtaining Equations (3) and (4):

(3) $$\varepsilon \prime_{rf}={\left[\frac{\rho_{f}}{\rho_{m}}\left(\sqrt{\varepsilon \prime_{rm}}-1\right)+1\right]}^{2}$$

(4) $$\varepsilon {\prime \prime }_{rf}={\left(\frac{\rho_{f}}{\rho_{m}}\right)}^{2}\varepsilon {\prime \prime }_{rm}$$

where $\varepsilon \prime_{rf}$ and ${\varepsilon \prime \prime }_{f}$ are the dielectric constant and the loss factor of the fabrics without the influence of air gaps, respectively, and $\varepsilon \prime_{rm}$ and ${\varepsilon \prime \prime }_{rm}$ are the dielectric constant and loss factor of the fabric-air aggregate, respectively.

### 2.6. Uncertainty Calculation

According to [33], the experimental standard uncertainty (*u*) of the arithmetic mean of the measurements was calculated as follows:

(5) $$u=\frac{S_{d}}{\sqrt{n}}$$

where *S_d_* is the experimental standard deviation and *n* corresponds to the number of tests used to calculate the estimate of a measurement (dielectric constant or loss factor). In this study, each permittivity measurement was repeated three times (*n* = 3) before being averaged to produce the mean result. As a result, the standard deviation of the three averaged values was divided by $\sqrt{3}$ to calculate uncertainty bars, or shaded areas, around the averaged observations.

## 3. Results

In this section, permittivity measurements are shown in relation to temperature, density, and internal moisture content for a variety of commercial fabric aggregates, such as cotton, polyamide, and polyester, as well as a variety of binary aggregates of these materials. In each case, fitting equations that closely approximate those dependencies are also included. The proper use of the CRI mixing equation provides a temperature estimation of the permittivity of various commercial fabrics without the influence of air gaps.

### 3.1. Permittivity Measurements for Cotton, Polyamide and Polyester Aggregates

#### 3.1.1. Temperature Dependence

**Figure 3:**
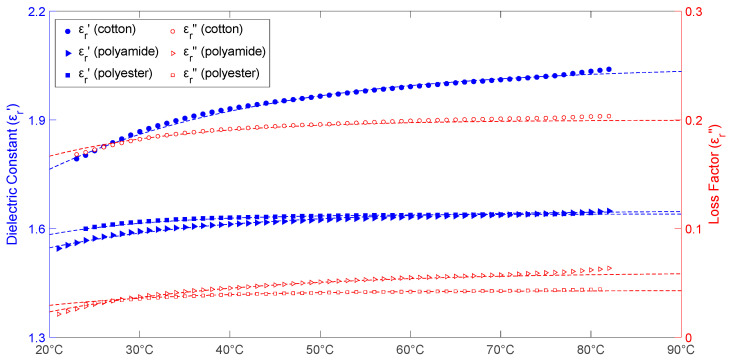
Temperature dependence of both dielectric constant (blue symbols) and loss factor (red symbols) measurements for cotton, polyamide and polyester samples. Fitted data from Equations (6)–(11) are represented by dashed lines; shaded areas reflect the measurement uncertainty.

Figure 3 depicts the temperature-dependent changes in relative permittivity of cotton, polyamide, and polyester aggregates. The cotton samples had moisture content around 6.1% and an apparent density of 0.37 g/cm^3,^ the polyamide samples had moisture content of 2.1% and an apparent density of 0.37 g/cm^3^, and the polyester samples had an apparent density of 0.375 g/cm^3^ and a moisture content of 0.75%. This figure also shows the data fitted to Equations (6)–(11) and the uncertainty in the measurements.

According to the results in Figure 3, cotton, polyamide, and polyester aggregates have fairly comparable temperature responses. All of them show increased dielectric constant and loss factor when the sample temperature rises. Cotton always has a higher dielectric constant and loss factor value, which could be related to the samples’ higher moisture content during tests. Cotton’s dielectric constant increases by more than 14% and its loss factor increases by more than 21.4% when the temperature rises from 24 to 77 °C. The observed results for the polyamide samples show 161% increases in loss factor and 6% increases in dielectric constant. In the examined temperature range, the dielectric constant rises by 3.4% and the loss factor rises by 54.5% for the polyester samples. Both the dielectric constant and the loss factor exhibit a similar pattern of growth with temperature: their values increase between 20 and 40 °C and then start to stabilize.

MATLAB’s Curve Fitting Tool was used to represent the temperature behavior of cotton, polyamide, and polyester aggregate permittivity as a function of temperature. The exponential fitting equations for the dielectric constant and loss factor of these aggregates are shown in Equations (6)–(11):

(6) $$\varepsilon_{r\_cotton}^{\prime }\left(T\right)=2.05-0.65e^{-0.041T}$$

(7) $$\varepsilon_{r\_cotton}^{\prime \prime }\left(T\right)=0.20-0.12e^{-0.064T}$$

(8) $$\varepsilon_{r\_polyamide}^{\prime }\left(T\right)=1.65-0.28e^{-0.05T}$$

(9) $$\varepsilon_{r\_polyamide}^{\prime \prime }\left(T\right)=0.06-0.09e^{-0.045T}$$

(10) $$\varepsilon_{r\_polyester}^{\prime }\left(T\right)=1.64-0.28e^{-0.08T}$$

(11) $$\varepsilon_{r\_polyester}^{\prime \prime }\left(T\right)=0.043-0.05e^{-0.065T}$$

where *T* denotes the aggregate’s temperature in degrees Celsius. The *R-square* and *RMSE* values for Equations (6)–(11) are shown in Table 2. For these fitting equations, the minimum *R-square* value was 0.9477 and the maximum *RMSE* value was 0.005, indicating an adequate fitting method.

#### 3.1.2. Bulk Density Dependence

**Figure 4:**
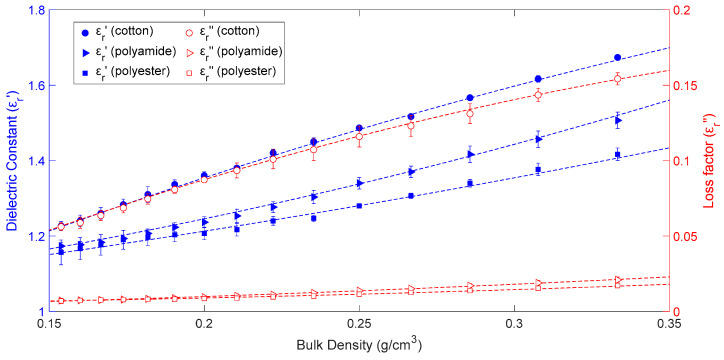
Dielectric constant (blue symbols) and loss factor (red symbols) bulk density dependence for cotton, polyamide, and polyester samples at 25 °C. Dashed lines represent fitted values obtained from Equations (12)–(17) and whiskers indicate uncertainty.

Figure 4 illustrates the measured and fitted evolution of permittivity versus sample bulk density of cotton, polyamide, and polyester aggregates. In all cases, the sample temperature was 25 °C. The moisture content of the cotton samples was 6.1%, while it was fixed at 2.1% for polyamide samples and 0.75% for polyester aggregates. As the data show, the dielectric constant and the loss factor both increase in value when the sample bulk density increases in all circumstances. The dielectric constant of cotton aggregates increased by 36.4% in this case, whereas the loss factor increased by 174.6%. Figure 4 shows a 29% increase in polyamide permittivity for the real part and an 188% increase for the imaginary part. The dielectric constant and loss factor increases in polyester aggregates were measured to be around 22.5 and 146%, respectively.

Since this type of quadratic behavior has been established in prior investigations, as detailed in [16], second-order polynomials have been used to model the density dependence of cotton, polyamide, and polyester aggregate permittivity under these conditions. Equations (12)–(17) indicate how cotton, polyamide, and polyester dielectric constants and loss factors vary with density:

(12) $$\varepsilon_{r\_cotton}^{\prime }\left(\rho \right)=-{2.64\rho }^{2}+3.75\rho +0.71$$

(13) $$\varepsilon_{r\_cotton}^{\prime \prime }\left(\rho \right)=-{0.94\rho }^{2}+\rho -0.075$$

(14) $$\varepsilon_{r\_polyamide}^{\prime }\left(\rho \right)={2.49\rho }^{2}+0.73\rho +1$$

(15) $$\varepsilon_{r\_polyamide}^{\prime \prime }\left(\rho \right)={0.107\rho }^{2}+0.028\rho \;$$

(16) $$\varepsilon_{r\_polyester}^{\prime }\left(\rho \right)={1.17\rho }^{2}+0.83\rho +1$$

(17) $$\varepsilon_{r\_polyester}^{\prime \prime }\left(\rho \right)={0.103\rho }^{2}-0.004\rho +0.004$$

where *ρ* is the bulk or apparent density of the aggregates expressed in g/cm^3^.

The *R-square* and *RMSE* values for Equations (12)–(17) are shown in Table 3. The minimum R-square value was 0.9928 and the maximum RMSE value was 0.0071, indicating again a satisfactory fitting procedure.

#### 3.1.3. Moisture Content Dependence

**Figure 5:**
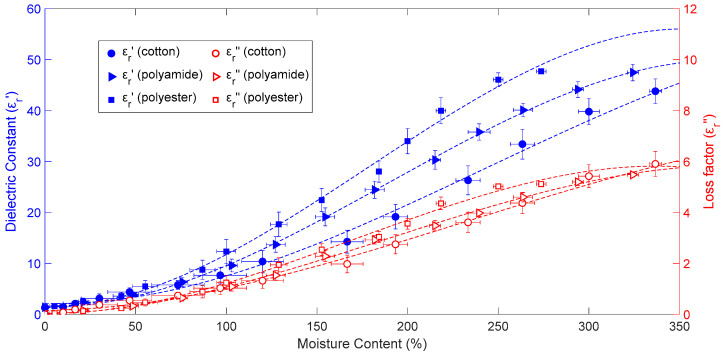
Variation in aggregate permittivity of cotton, polyamide, and polyester with moisture content at 25 °C and a starting bulk density of 0.98 g/cm^3^. Equations (18)–(23) were used to calculate the fitted values, which are represented by dashed lines; each measurement’s uncertainty is shown by whiskers.

Figure 5 depicts the link between permittivity changes and dry-basis moisture content in cotton, polyamide, and polyester samples. The measurements were carried out at a sample temperature of 25 °C and an initial bulk density of 0.98 g/cm3, which corresponded to the maximum value of sample moisture content. The fitted values obtained with Equations (18)–(23) are represented by dashed lines and the measurement uncertainty is indicated by whiskers. The data in Figure 5 leads to the conclusion that all fabrics’ dielectric constant and loss factor values increase as moisture content rises, though this increment is not constant. In fact, this increase is more noticeable in all cases for moisture ranges between 70 and 250%. Due to the bonding between moisture and fabric fibers, which prevents water from moving freely, the increment is lower for moisture contents under 70%. More free water is present in the fabrics above a moisture content of 70%, so permittivity rises more rapidly as moisture content levels rise. At high moisture content levels, polyamide and polyester have greater dielectric constant values than cotton, indicating that cotton has a more pronounced hygroscopic activity than the other materials. At moisture content levels less than 70%, cotton has greater dielectric constant and loss factor values than polyamide and polyester.

Equations (18)–(23) show third-order polynomial fits for the dielectric constant and loss factor of cotton, polyamide, and polyester aggregates versus moisture content, which were the functions with more effective fitting results than second-order polynomials and which were used in previous works [26]:

(18) $$\varepsilon_{r\_cotton}^{\prime }\left(X\right)=-X^{3}+7.28X^{2}-0.87X+2.13$$

(19) $$\varepsilon_{r\_cotton}^{\prime \prime }\left(X\right)=-0.13X^{3}+0.91X^{2}+0.09X+0.15$$

(20) $$\varepsilon_{r\_polyamide}^{\prime }\left(X\right)=-1.94X^{3}+11.857X^{2}-1.09X+1.58$$

(21) $$\varepsilon_{r\_polyamide}^{\prime \prime }\left(X\right)=-0.21X^{3}+1.18X^{2}+0.08X+0.02$$

(22) $$\varepsilon_{r\_polyester}^{\prime }\left(X\right)=-2.59X^{3}+13.85X^{2}-1.23X+1.70$$

(23) $$\varepsilon_{r\_polyester}^{\prime \prime }\left(X\right)=-0.31X^{3}+1.58X^{2}-0.09X+0.04$$

where *X* represents the average dry-basis moisture content, as indicated in Equation (1), of each measurement. Table 4 displays the *R-square* and *RMSE* values for Equations (18)–(23). Again, demonstrating a successful fitting method, the minimum *R-square* value was 0.9959 and the maximum *RMSE* value was 1.0249.

### 3.2. Permittivity Measurement for Binary Fabrics Aggregates

This section shows and discusses the permittivity behavior of various binary combinations of cotton, polyamide, and polyester aggregates in relation to temperature, density, moisture content, and aggregate composition.

#### 3.2.1. Temperature Dependence

**Figure 6:**
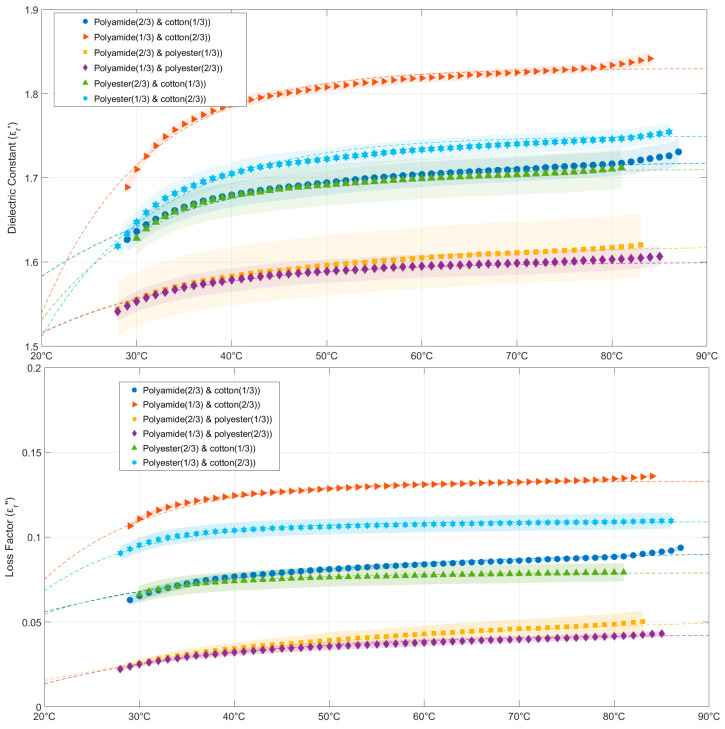
Dielectric constant (**above**) and loss factor (**below**) evolution of cotton, polyamide, and polyester binary aggregates versus sample temperature and fabric percentages. Symbols represent experimental data; dashed lines show data fitted with Equations (24)–(35) and colored areas show measurements’ uncertainty. Measurement conditions: cotton-polyamide aggregates (sample bulk density = 0.31 g/cm^3^, average moisture content ranging from 3.31% to 4.71%); cotton-polyester aggregates (sample bulk density = 0.31 g/cm^3^, average moisture content ranging from 2.45% to 4.27%); polyamide-polyester aggregates (sample bulk density = 0.31 g/cm^3^, average moisture content ranging from 1.16 to 1.58%).

Figure 6 provides both measurements and data fitted to Equations (24)–(35) to show how temperature and the amount of fabric mass in each aggregate affect the permittivity of binary combinations of cotton, polyamide, and polyester aggregates. According to the data, all fabric aggregates increase both the loss factor and dielectric constant with rising temperatures, as would be expected based on the results of the single fabric aggregates shown in the previous section. Fabric aggregates with larger percentages of cotton have higher dielectric constants and loss factor values due to their higher moisture content, while polyester and polyamide binary aggregates exhibit the lowest permittivity values. For these binary aggregates, the loss factor and dielectric constant stabilize above 40 °C, as anticipated from the data in Figure 3. Equations (24)–(35) represent the exponential fits for cotton, polyamide, and polyester binary aggregates’ dielectric constant and loss factor as a function of sample temperature:

(24) $$\varepsilon_{r_{c33}\%{\_}_{a67}\%}^{\prime }\left(T\right)=1.72-0.42e^{-0.056T}$$

(25) $$\varepsilon_{r_{c33}\%{\_}_{a67}\%}^{\prime \prime }\left(T\right)=0.092-0.08e^{-0.04T}$$

(26) $$\varepsilon_{r_{c67}\%{\_}_{a33}\%}^{\prime }\left(T\right)=1.83-1.87e^{-0.093T}$$

(27) $$\varepsilon_{r_{c67}\%{\_}_{a33}\%}^{\prime \prime }\left(T\right)=0.133-0.35e^{-0.09T}$$

(28) $$\varepsilon_{r_{e33}\%{\_}_{a67}\%}^{\prime }\left(T\right)=1.62-0.28e^{-0.05T}$$

(29) $$\varepsilon_{r_{e33}\%{\_}_{a67}\%}^{\prime \prime }\left(T\right)=0.054-0.07e^{-0.03T}$$

(30) $$\varepsilon_{r_{e67}\%{\_}_{a33}\%}^{\prime }\left(T\right)=1.60-0.31e^{-0.065T}$$

(31) $$\varepsilon_{r_{e67}\%{\_}_{a33}\%}^{\prime \prime }\left(T\right)=0.043-0.08e^{-0.05T}$$

(32) $$\varepsilon_{r_{e33}\%{\_}_{c67}\%}^{\prime }\left(T\right)=1.75-1.23e^{-0.082T}$$

(33) $$\varepsilon_{r_{e33}\%{\_}_{c67}\%}^{\prime \prime }\left(T\right)=0.109-0.30e^{-0.10T}$$

(34) $$\varepsilon_{r_{e67}\%{\_}_{c33}\%}^{\prime }\left(T\right)=1.71-0.96e^{-0.084T}$$

(35) $$\varepsilon_{r_{e67}\%{\_}_{c33}\%}^{\prime \prime }\left(T\right)=0.079-0.12e^{-0.08T}$$

where the subscripts *c*, *a*, and *e* stand for cotton, polyamide, and polyester, respectively, while the percentages represent the mass content of each aggregate. Table 5 shows the *R-square* and *RMSE* values for Equations (24)–(35) with a minimum *R-square* value of 0.9608 and a maximum *RMSE* value of 0.0057.

#### 3.2.2. Bulk Density Dependence

**Figure 7:**
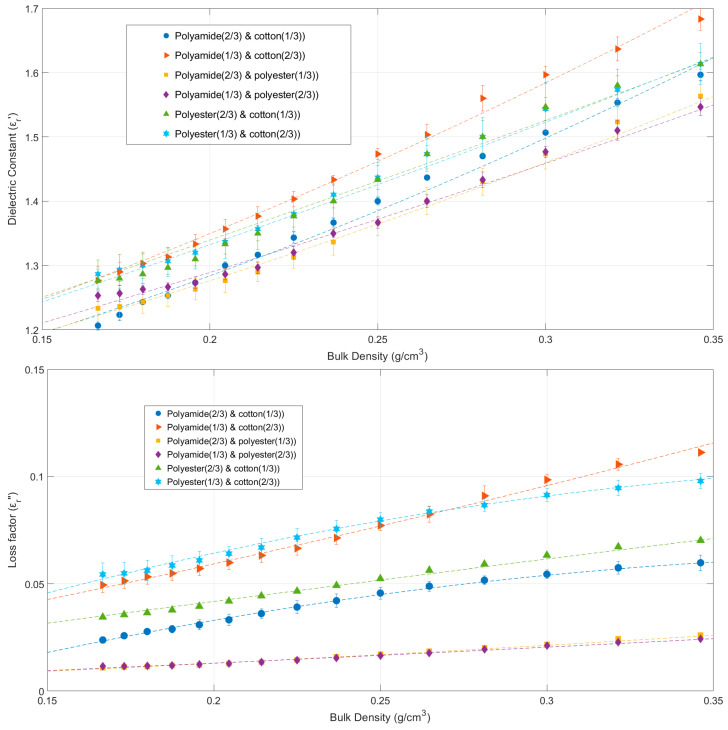
Dielectric constant (**above**) and loss factor (**below**) variation of cotton, polyamide, and polyester binary aggregates versus sample bulk density and fabric percentages. Symbols represent experimental data; dashed lines show data fitted with Equations (24)–(35) and whiskers show measurement uncertainty. Measurement conditions: cotton-polyamide aggregates (average moisture content ranging from 3.31 to 4.71%); cotton-polyester aggregates (average moisture content ranging from 2.45 to 4.27%); polyamide-polyester aggregates (average moisture content ranging from 1.16 to 1.58%). Sample temperature was 25 °C in all cases.

Figure 7 shows how density and the percentage of fabric mass affect the permittivity of cotton, polyamide, and polyester binary aggregates. This figure includes the fitted results using Equations (36)–(47) as well. According to the data, all binary aggregates increase both the dielectric constant and the loss factor with increasing bulk density values, as would be expected based on the results of the single aggregates presented in Section 3.1.

Again, binary aggregates with a higher proportion of cotton have higher values for the dielectric constant and the loss factor. The lowest permittivity values, on the other hand, are produced by polyamide and polyester binary aggregates. The loss factor and dielectric constant variation of cotton, polyamide, and polyester binary aggregates as a function of sample bulk density (*ρ*) are fitted by second-order polynomials in Equations (36)–(47):

(36) $$\varepsilon_{r_{c33}\%{\_}_{a67}\%}^{\prime }\left(\rho \right)={2.40\rho }^{2}+0.94\rho +1$$

(37) $$\varepsilon_{r_{c33}\%{\_}_{a67}\%}^{\prime \prime }\left(\rho \right)=-{0.6\rho }^{2}+0.51\rho -0.045$$

(38) $$\varepsilon_{r_{c67}\%{\_}_{a33}\%}^{\prime }\left(\rho \right)={1.99\rho }^{2}+1.35\rho +1$$

(39) $$\varepsilon_{r_{c67}\%{\_}_{a33}\%}^{\prime \prime }\left(\rho \right)={0.23\rho }^{2}+0.25\rho \;$$

(40) $$\varepsilon_{r_{e33}\%{\_}_{a67}\%}^{\prime }\left(\rho \right)={1.51\rho }^{2}+1.08\rho +1$$

(41) $$\varepsilon_{r_{e33}\%{\_}_{a67}\%}^{\prime \prime }\left(\rho \right)={0.064\rho }^{2}+0.052\rho \;$$

(42) $$\varepsilon_{r_{e67}\%{\_}_{a33}\%}^{\prime }\left(\rho \right)={0.83\rho }^{2}+1.28\rho +1$$

(43) $$\varepsilon_{r_{e67}\%{\_}_{a33}\%}^{\prime \prime }\left(\rho \right)={0.031\rho }^{2}+0.059\rho \;$$

(44) $$\varepsilon_{r_{e33}\%{\_}_{c67}\%}^{\prime }\left(\rho \right)={0.54\rho }^{2}+1.59\rho +1$$

(45) $$\varepsilon_{r_{e33}\%{\_}_{c67}\%}^{\prime \prime }\left(\rho \right)=-{0.68\rho }^{2}+0.607\rho -0.03$$

(46) $$\varepsilon_{r_{e67}\%{\_}_{c33}\%}^{\prime }\left(\rho \right)={0.81\rho }^{2}+1.50\rho +1$$

(47) $$\varepsilon_{r_{e67}\%{\_}_{c33}\%}^{\prime \prime }\left(\rho \right)=-{0.04\rho }^{2}+0.217\rho \;$$

The *R-square* and *RMSE* values for Equations (36)–(47) are displayed in Table 6. Every fitting equation yields *R-square* values greater than 0.9878, while *RMSE* is always less than 0.0135.

#### 3.2.3. Moisture Content Dependence

**Figure 8:**
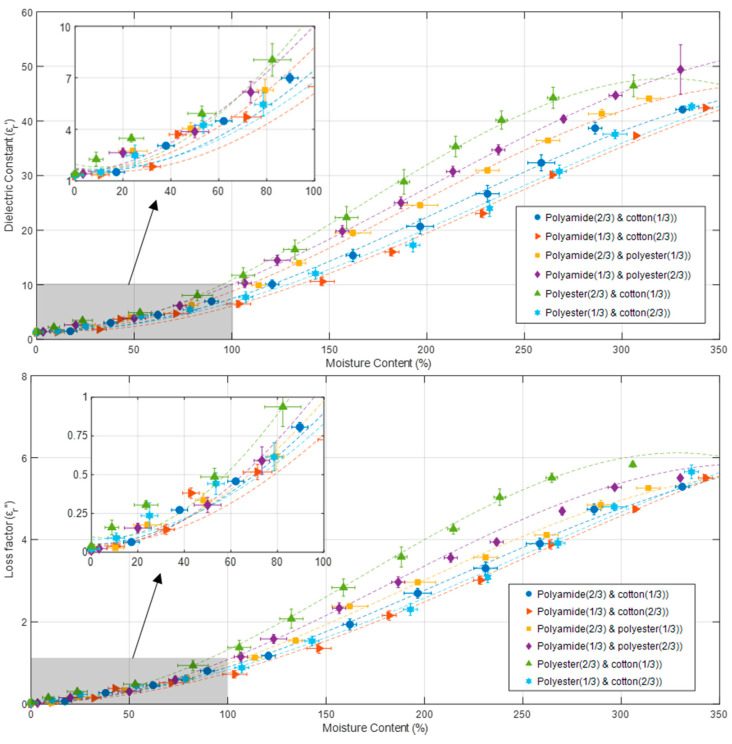
Dielectric constant (**above**) and loss factor (**below**) variation of cotton, polyamide, and polyester binary aggregates versus sample moisture content and fabric percentages. Symbols represent experimental data; dashed lines show data fitted with Equations (48)–(59) and whiskers show measurements’ uncertainty. Sample temperature and initial sample density was 25 °C and 0.98 g/cm^3^, respectively, for all measurements.

Figure 8 shows how the moisture content (dry basis) and the proportion of fabric mass influence the permittivity of cotton, polyamide, and polyester binary aggregates, and plots the data fitted to Equations (48)–(59) with dashed lines. According to the data, all fabric aggregates increase both the dielectric constant and the loss factor with increasing moisture content, as expected. Figure 8 exhibits a similar pattern to that shown in Figure 5 for single aggregates: for moisture contents less than 70%, permittivity increases with increasing moisture content at a slower rate than the increments seen in the range of 70 to 250% moisture content. The permittivity rises at a slower rate for moisture content greater than 250%.

Equations (48)–(59) show the third-order polynomial fits, which again were the functions with best fitting performance, for the dielectric constant and the loss factor of cotton, polyamide, and polyester binary aggregates as a function of sample dry-basis moisture content (X):

(48) $$\varepsilon_{r_{c33}\%{\_}_{a67}\%}^{\prime }\left(X\right)=-1.38X^{3}+8.70X^{2}-1.51X+1.65$$

(49) $$\varepsilon_{r_{c33}\%{\_}_{a67}\%}^{\prime \prime }\left(X\right)=-0.15X^{2}+0.96X^{2}+0.04X+0.05$$

(50) $$\varepsilon_{r_{c67}\%{\_}_{a33}\%}^{\prime }\left(X\right)=-1.09X^{3}+7.75X^{2}-2.25X+1.70$$

(51) $$\varepsilon_{r_{c67}\%{\_}_{a33}\%}^{\prime \prime }\left(X\right)=-0.11X^{3}+0.84X^{2}-0.02X+0.05$$

(52) $$\varepsilon_{r_{e33}\%{\_}_{a67}\%}^{\prime }\left(X\right)=-1.83X^{3}+10.49X^{2}-1.63X+1.75$$

(53) $$\varepsilon_{r_{e33}\%{\_}_{a67}\%}^{\prime \prime }\left(X\right)=-0.20X^{3}+1.15X^{2}-0.01X+0.04$$

(54) $$\varepsilon_{r_{e67}\%{\_}_{a33}\%}^{\prime }\left(X\right)=-1.55X^{3}+9.19X^{2}+0.98X+1.45$$

(55) $$\varepsilon_{r_{e67}\%{\_}_{a33}\%}^{\prime \prime }\left(X\right)=-0.25X^{3}+1.37X^{2}-0.08X+0.04$$

(56) $$\varepsilon_{r_{e33}\%{\_}_{c67}\%}^{\prime }\left(X\right)=-1.03X^{3}+7.30X^{2}-1.22X+1.73$$

(57) $$\varepsilon_{r_{e33}\%{\_}_{c67}\%}^{\prime \prime }\left(X\right)=-0.11X^{3}+0.83X^{2}+0.03X+0.08$$

(58) $$\varepsilon_{r_{e67}\%{\_}_{c33}\%}^{\prime }\left(X\right)=-3.02X^{3}+15.12X^{2}-3.15X+1.97$$

(59) $$\varepsilon_{r_{e67}\%{\_}_{c33}\%}^{\prime \prime }\left(X\right)=-0.36X^{3}+1.81X^{2}-0.23X+0.10$$

where *X* represents the average dry-basis moisture content, as indicated in Equation (1), of each measurement. Table 7 indicates the fitting results for Equations (48)–(59). *R-square* values higher than 0.9954 and *RMSE* values lower than 1.0692 are obtained by these fitting equations.

### 3.3. Estimation of Fabrics Permittivity versus Temperature Calculated Using CRI Model for Dielectric Mixtures

**Figure 9:**
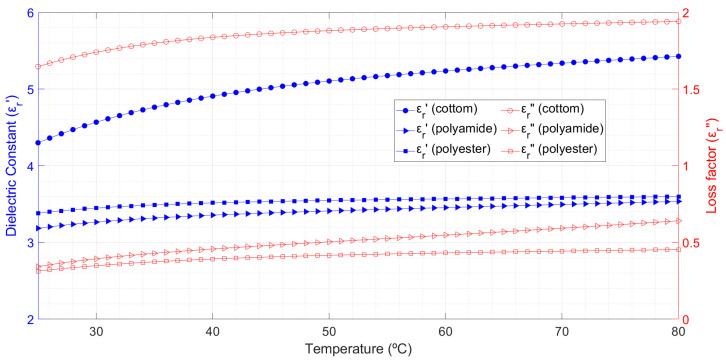
Permittivity estimation of cotton, polyamide, and polyester fabrics versus their temperatures without the influence of air. Calculation carried out using CRI model for dielectric mixtures and Equations (6)–(11). Moisture content: cotton (6.1%), polyamide (2.1%), and polyester (0.75%).

Using the CRI dielectric mixture model to estimate the fabric permittivity without the influence of air gaps, as stated in Section 2.5, Figure 9 illustrates the evolution of the permittivity of cotton, polyamide, and polyester fabrics as a function of their temperature. Since the fitting procedure yielded high *R-square* values, Equations (6)–(11), rather than the experimental data, were used for this estimation. The fabric density values used in CRI calculations for cotton, polyamide, and polyester were 1.14, 1.14, and 1.23 g/cm^3^, respectively [29,30,31].

The results show that, as predicted by the CRI model equations, higher values are achieved for the pure fabrics than for the aggregates, which include air in the permittivity estimation. All fabrics demonstrate that as temperature rises, the dielectric constant and the loss factor both rise as well. It’s also noteworthy that cotton has a substantially higher loss factor and dielectric constant than polyester and polyamide due to its higher moisture content during the measurements.

## 4. Discussion and Conclusions

This study describes and applies a novel approach for the characterization of permittivity of cotton, polyamide, and polyester textile aggregates in varied fabric composition, temperature, bulk density, and moisture content ranges around the 2.45 GHz ISM band. When these characteristics increase, the permittivity values increase in all circumstances, both for pure fabric aggregates and for binary combinations of cotton, polyamide, and polyester aggregates.

Exponential functions were successfully employed to predict the general pattern of observations for the rise of both the dielectric constant and the loss factor versus aggregate temperature, which showed an initial increase followed by stabilization for temperature values above 40 °C in most cases. For bulk density and moisture content evolution, second- and third-order polynomials accurately represented the observed permittivity trend. As previously stated, the use of second-order polynomials in density dependence measurements was motivated by prior studies that revealed this dependence. However, for moisture content dependence measurements, an additional order in polynomials was required because second-order polynomials did not adequately match the experimental data.

By far the most influential variable on permittivity changes is moisture content, which causes considerable variation in both the dielectric constant and the loss factor of pure fabric and aggregate samples. Cotton samples, for example, exhibit a change in the dielectric constant and loss factor of roughly 259 and 564% when the moisture content of the sample increases from 0 to 100%. These differences are substantially larger for polyamide and polyester, showing that cotton has stronger hygroscopic behavior. Permittivity increases more slowly for all fabric aggregates with moisture content less than 70%, indicating that below this threshold, water is bonded to the fabric fibers and has less mobility when the microwave electric field is applied.

This wide range of permittivity versus moisture content for these textile aggregates should be taken into account when building microwave dryers or resonant devices, given the fact that they would operate under a wide range of dielectric constant and loss factor values. Sweating, for example, should be taken into account when designing and building sensors or smart antennas for sportswear. Microwave sources for microwave dryers should also have low reflection coefficient values when working with loads that have a large permittivity change.

Finally, the loss factor of cotton fabrics is higher than that of polyamide and polyester fabrics for moisture content less than 70%, particularly in the 0 to 20% moisture content range. This indicates that, due to selective heating, within that moisture content range cotton will dry faster than polyamide and polyester in textile aggregates during microwave drying processes, which may be a problem because uniform final moisture content levels are expected regardless of fabric type. Further research is envisaged in this direction.

## Figures and Tables

**Table 1:** Mass percentage of fabrics for each aggregate specimen.

Specimen	Cotton (%)	Polyester (%)	Polyamide (%)
1	100	0	0
2	0	100	0
3	0	0	100
4	67	33	0
5	33	67	0
6	67	0	33
7	33	0	67
8	0	67	33
9	0	33	67

**Table 2:** *R-square* and *RMSE* values for Equations (6)–(11).

Fitting Equation	*R-Square*	*RMSE*
(6)	0.9940	0.0050
(7)	0.9477	0.0020
(8)	0.9816	0.0033
(9)	0.9723	0.0017
(10)	0.9572	0.0020
(11)	0.9719	0.0005

**Table 3:** *R-square* and *RMSE* values for Equations (12)–(17).

Fitting Equation	*R-Square*	*RMSE*
(12)	0.9989	0.0046
(13)	0.9980	0.0014
(14)	0.9955	0.0070
(15)	0.9944	0.0003
(16)	0.9936	0.0071
(17)	0.9928	0.0003

**Table 4:** *R-square* and *RMSE* values for Equations (18)–(23).

Fitting Equation	*R-Square*	*RMSE*
(18)	0.9959	1.0249
(19)	0.9964	0.1318
(20)	0.9996	0.3330
(21)	0.9989	0.0655
(22)	0.9966	0.9561
(23)	0.9960	0.1164

**Table 5:** *R-square* and *RMSE* values for Equations (24)–(35).

Fitting Equation	*R-Square*	*RMSE*
(24)	0.9723	0.0037
(25)	0.9694	0.0012
(26)	0.9778	0.0048
(27)	0.9623	0.0012
(28)	0.9913	0.0017
(29)	0.9788	0.0010
(30)	0.9608	0.0030
(31)	0.9816	0.0007
(32)	0.9635	0.0057
(33)	0.9663	0.0008
(34)	0.9606	0.0037
(35)	0.9808	0.0004

**Table 6:** *R-square* and *RMSE* values for Equations (36)–(47).

Fitting Equation	*R-Square*	*RMSE*
(36)	0.9908	0.0114
(37)	0.9972	0.0006
(38)	0.9937	0.0103
(39)	0.9943	0.0015
(40)	0.9927	0.0101
(41)	0.9907	0.0005
(42)	0.9906	0.0104
(43)	0.9888	0.0005
(44)	0.9928	0.0100
(45)	0.9947	0.0011
(46)	0.9878	0.0135
(47)	0.9895	0.0013

**Table 7:** *R-square* and *RMSE* values for Equations (48)–(59).

Fitting Equation	*R-Square*	*RMSE*
(48)	0.9971	0.8515
(49)	0.9984	0.0795
(50)	0.9973	0.8172
(51)	0.9987	0.0769
(52)	0.9988	0.5594
(53)	0.9975	0.0985
(54)	0.9995	0.3807
(55)	0.9967	0.1131
(56)	0.9954	1.0692
(57)	0.9967	0.1210
(58)	0.9981	0.7107
(59)	0.9981	0.0917

## Data Availability

Not applicable.

## Abbreviations

The following abbreviations are used in this manuscript:

CRI	Complex Refractive Index
DKV	Dielectric Kit for Vials
$\varepsilon \prime_{m}$	dielectric constant the fabrics-air aggregate
$\varepsilon_{r}^{\prime }$	dielectric constant
$\varepsilon \prime_{rf}$	dielectric constant of the fabrics without the influence of air gaps
$\varepsilon_{r}^{\prime \prime }$	loss factor
${\varepsilon \prime \prime }_{rf}$	loss factor of the fabrics without the influence of air gaps
${\varepsilon \prime \prime }_{rm}$	loss factor of the fabrics-air aggregate
$\varepsilon_{ra}$	air relative permittivity
$\varepsilon_{rf}$	permittivity of the cotton, polyester, or polyamide fabrics without the influence of air gaps
$\varepsilon_{rm}$	relative permittivity of the air-fabrics aggregate,
ISM	Industrial, Scientific and Medical
ITACA	Institute of Information and Communication Technologies
*m_d_*	dry mass of samples (g)
*m_w_*	mass of samples containing moisture (g)
*n*	number of tests used to calculate the estimate of the dielectric constant or loss factor
*ρ*	density of samples (g/cm^3^)
$\rho_{m}$	bulk density of fabrics’ aggregates (g/cm^3^)
$\rho_{f}$	density of fabrics (g/cm^3^)
*RMSE*	Root Mean Squared Error
*R-square*	square of the correlation between the observed values and the predicted response values
*S_d_*	experimental standard deviation
*T*	temperature of samples (°C)
*u*	uncertainty of the measurements
$v_{a}$	volume fraction of air
$v_{f}$	volume fraction of fabrics
*X*	moisture content (dry basis)
